# Dual-core-component multiphasic bioceramic granules with selective-area porous structures facilitating bone tissue regeneration and repair

**DOI:** 10.1039/d4ra00911h

**Published:** 2024-04-02

**Authors:** Binji Cao, Lijun Xie, Yan Xu, Jian Shen, Yan Zhang, Yingjie Wang, Xisheng Weng, Zhaonan Bao, Xianyan Yang, Zhongru Gou, Cong Wang

**Affiliations:** a Department of Orthopedic Surgery, The Second Affiliated Hospital, Zhejiang University School of Medicine #88 Jiefang Road Hangzhou 310009 Zhejiang Province China 05wangcong@zju.edu.cn; b Bio-nanomaterials and Regenerative Medicine Research Division, Zhejiang-California International Nanosystem Institute, Zhejiang University Hangzhou 310058 China zhrgou@zju.edu.cn (+86) 571-8697 1539 (+86) 571-8820 8353; c Department of Emergency Medicine, The Second Affiliated Hospital, Zhejiang University School of Medicine and Institute of Emergency Medicine #88 Jiefang Road Hangzhou 310009 Zhejiang Province China; d Department of Orthopedic Surgery, State Key Laboratory of Complex Severe and Rare Diseases, Peking Union Medical College Hospital, Chinese Academy of Medical Science and Peking Union Medical College Beijing 100730 China

## Abstract

Ca-phosphate/-silicate ceramic granules have been widely studied because their biodegradable fillers can enhance bone defect repair accompanied with bioactive ion release and material degradation; however, it is a challenge to endow bioceramic composites with time-dependent ion release and highly efficient osteogenesis *in vivo*. Herein, we prepared dual-core-type bioceramic granules with varying chemical compositions beneficial for controlling ion release and stimulating osteogenic capability. Core–shell-structured bioceramic granules (P8-Sr4@Zn3, P8-Sr4@TCP, and P8-Sr4@HAR) composed of 8% P- and 4% Sr-substituting wollastonite (P8, Sr4) dual core components and different shell components, such as 3% Zn-substituting wollastonite (Zn3), β-tricalcium phosphate (β-TCP), and hardystonite (HAR), were prepared by cutting extruded core–shell fibers through dual-core ternary nozzles, followed by high-temperature sintering post-treatment. The experimental results showed that nonstoichiometric wollastonite core components contributed to more biologically active ion release in Tris buffer *in vitro*, and the sparingly dissolvable shell component readily maintained the granule morphology *in vivo*; thus, such bioceramic implants can adjust new bone growth and material degradation over time. In particular, bioceramic granules encapsulated by the TCP shell exhibited the most appreciable osteogenic capacity and expected biodegradation, which was mostly favorable for bone repair in critical bone defects. It is reasonable to consider that this new multiphasic bioceramic granule design is versatile for developing next-generation implants for various bone damage repairs.

## Abstract 2

Ca-phosphate/-silicate ceramic granules have been widely studied because their abilities of biodegradable, bioactive ion release and material degradation; however, it is difficult to synergise time-dependent ion release and highly efficient osteogenesis *in vivo*.

## Graphical abstract

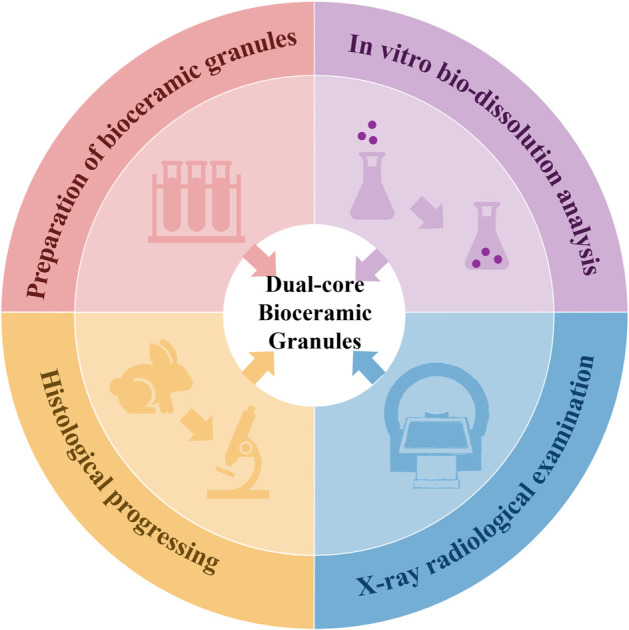

## 1. Introduction

Bone is a type of self-remodeling, self-repairing, dynamic vascularized tissue; thus, slight bone damage is restorable without surgical intervention. However, critical size bone fractures or defects are usually require intervention by orthotopic extraosseous implants to achieve bone damage healing.^1,2^ Nowadays, more and more artificial fillers have been developed to accelerate damaged bone repair and recover anatomical structures and mechanical functions. In this aspect, various biodegradable ceramics have been extensively investigated because of their unique features and prospective purposes in the field of bone regenerative medicine.^3,4^ It is well known that many Ca-phosphate (CaP) ceramics, such as hydroxyapatite (HA) and β-tricalcium phosphate (β-TCP) and their biphasic composites, possess excellent biocompatibility, biodegradability, and bioactivity.^5^ These characteristics make them promising candidate materials for promoting tissue regeneration and preventing delayed healing. In the past two decades, however, the clinical applications of CaP bioceramics have demonstrated that these conventional bioceramics are suboptimal for bone defect repair, mainly, owing to their low bioactivity and unexpected biodegradability.^6–9^ Therefore, it is still a challenge to develop bioceramic implants with tailorable biological performance to promote bone regeneration and repair in the clinic.

However, some Ca- or Ca-(Mg, Zn)-silicate ceramics, including wollastonite (CaSiO_3_ and CSi), bredigite (Ca_7_Mg(SiO_4_))_4_, akermanite (Ca_2_MgSi_2_O_7_), and hardystonite (Ca_2_ZnSi_2_O_7_), have been investigated as granules or scaffolds in the field of regenerative medicine and dentistry.^10–12^ It is widely agreed that these silicate bioceramics are bioactive and exhibit appreciable osteostimulative efficacy by stimulating osteogenic stem cell proliferation, differentiation, and vascularization.^13–16^ Unfortunately, the uncontrollable biodegradable rate and ion release dosage (affecting osteogenic activity) of Ca-(Mg, Zn)-silicate ceramics have restricted their applications as bone-repair fillers. For instance, hardystonite (HAR) has been highly appreciated for its potential in bone repair due to its appreciable contact-antibacterial potential and supporting osteogenic cell attachment and proliferation,^17^ whereas its shortcoming lies in its sparingly dissolvable behavior and poor biodegradable kinetics in the living body. In contrast, the pure wollastonite (CSi) bioceramic exhibits too fast a biodegradable *in vivo* and its porous scaffold is suboptimal for completing bone repair in critical bone defect.^18^ In this respect, our team has developed a series of foreign ion doping routes and core–shell structure designs to tune the biological performances of the CSi-based bioceramic implants.^19–21^ Previous studies have indicated that the selective foreign ion doping route could adjust the sintering and mechanical properties of CSi, especially dilute Mg doping into CSi bioceramic, which could produce outstanding compressive and flexible resistance.^22^ Additionally, Sr doping CSi could tune the biodegradation of CSi bioceramics and endow more biological performances in the bone defect repair area.^16,23^ In fact, P is thought to be a biologically essential ion that could promote osteoblastic cell proliferation,^24,25^ and our recent study also implied that 8% P and 3% Zn doping may accelerate or retard biomimetic apatite re-mineralization of CSi bioceramics *in vitro*, especially the latter could inhibit bio-dissolution of CSi ceramic *in vitro* (not published). In fact, the choice to control the ion release of the bioceramic composite and impair the biodegradation kinetics of the entire biomaterial by increasing one component while decreasing the other is suboptimal.^26–28^ Hence, it is difficult to fabricate composite biomaterials that may produce finely tuned release behavior of specific doping ions free of potential influence by the other bioceramic components in the composites.

Based on the aforementioned concerns, herein, we aimed to extrapolate our core-shell-structured granule design and develop dual-core-type core–shell bioceramic granules with a dual ion doping dual-core component using ternary nozzle equipment. The component selections in the dual-core-type granule were inspired by porous structure support for long-term bone repair requirements. Accordingly, the sparingly dissolvable HAR, β-TCP and 3% Zn-doped were used as the shell components to facilitate bone formation in the closely packed bioceramic granule implants. The component distribution, ion release behavior and osteogenic response in femoral bone defects for the multiphasic bioceramic granules were evaluated systematically. The experimental findings confirmed our hypothesis that the finely controlled component distribution and ion release in such dual-core bioceramic granules could produce different ion release dosages and bone regeneration rates, thus helping to develop the new bioceramic composites and expected biological performances in bone regenerative medicine.

## 2. Materials and methods

### 2.1 Materials and reagents

All the inorganic salt reagents, carboxylate chitosan (CC; degree of deacetylation >90%) and carboxymethyl cellulose (CMC) were purchased from Sinopharm Chemical Reagent Co., China. Trihydroxymethyl aminomethane (Tris) and phosphate buffer solution (PBS) were obtained from Sigma-Aldrich (St. Louis, MO, USA). The reagents were used directly without further purification.

### 2.2 Preparation of bioceramic powders

The 4 mol% Sr, 8 mol% P, and 3 mol% Zn-doping CSi (denoted as Sr4, P8, and Zn3, respectively) powders were first synthesized by applying wet-chemical methods, as reported previously.^29^ The 8% trisodium silicate was replaced by diammonium hydrogen phosphate during the preparation of the P8 powder, and 4% or 3% calcium nitrate was replaced by strontium nitrate or zinc nitrate for Sr4 and Zn3 powder synthesis, respectively. The β-TCP powder was prepared by a conventional co-precipitation route.^30^ The HAR powder was synthesized using a typical sol–gel method.^31^ These bioceramic powders were ball-milled down to an average particle size of less than 5 μm.

### 2.3 Preparation of dual-core multiphasic bioceramic granules

**Fig. 1:**
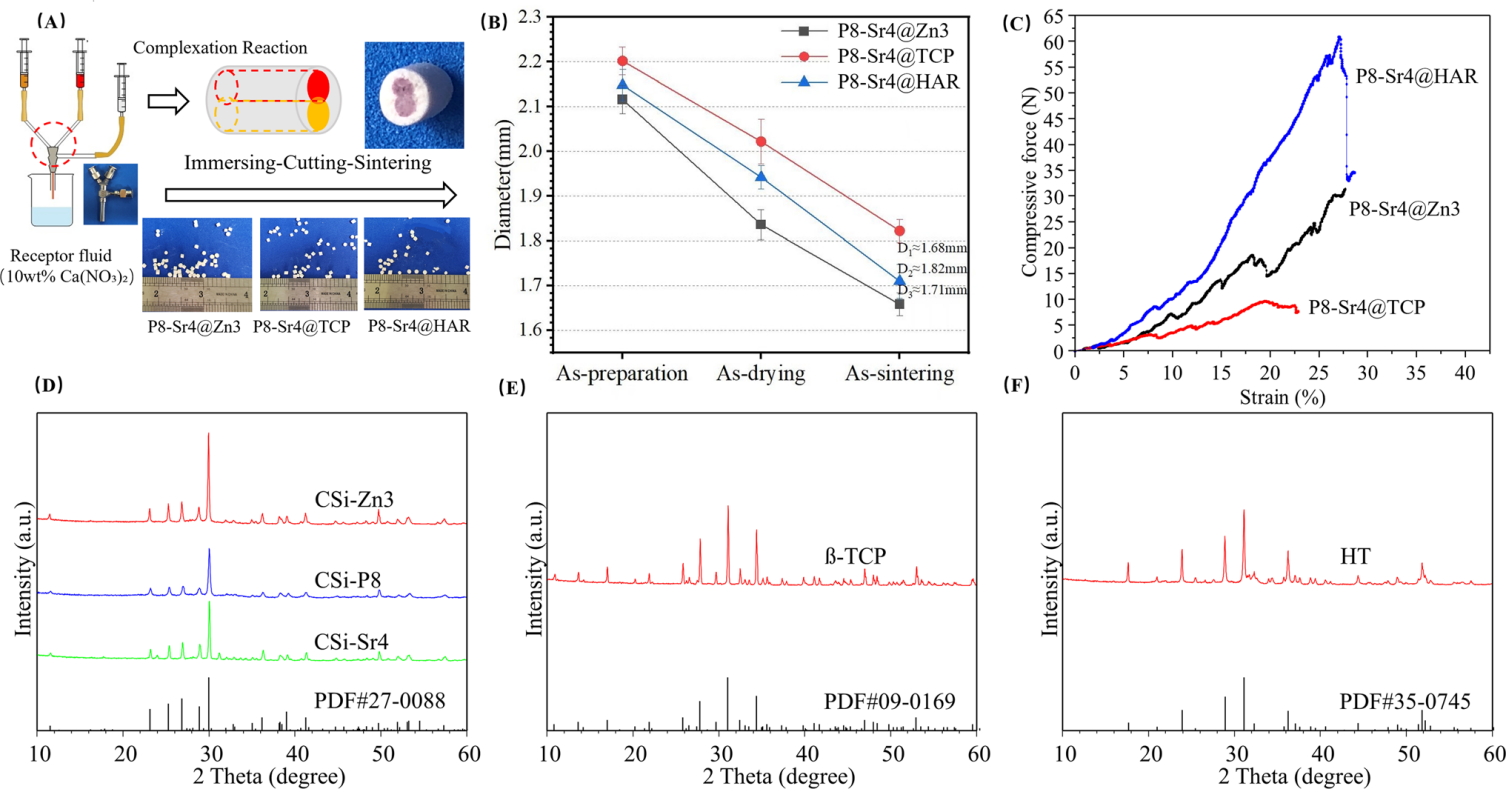
Schematic illustration of dual-core-type granule preparation and primary characterization of the core–shell bioceramic granules. (A) Schematic procedure of bioceramic fiber precursor preparation (inset shows the outward appearance of granules after sintering treatment); (B) changes in diameter of the fibrous granules; (C) representative stress–strain curves for the granules; (D) XRD patterns of ion-doped CSi powders; (E) XRD pattern of β-TCP powders; (F) XRD pattern of har powders.

Superfine powders were added to aqueous solutions composed of 7.0 wt% CC and 1.5 wt% CMC in a ratio of 2 : 2 : 1 in mass under magnetic stirring. HAR powder was also treated in the same way. The five groups of bioceramic slurries were magnetically agitated for 2 hours to form the core and shell slurries. After that, minor amounts of Sudan III were added to the P8 and Sr4 slurry and PMMA microspheres as porogens were added into the shell slurries with a PMMA/powder mass ratio of 28 : 72 before bioceramic fiber preparation as a coloring agent. The different slurries for the P8 and Sr4 dual-core components and Zn3, TCP and HAR shell components were transferred into two 20 mL syringes and fixed in the special ternary bilayer nozzle system (see Fig. 1A). The dual-core-type core–shell fibers with similar (inner/outer) diameter ratio were fabricated by extruding the slurries through three corresponding tunnels of 18G-18G@10G nozzles (Ø 0.8 mm, Ø 0.8 mm, Ø 2.50 mm in inner diameter), followed by 1.0 mol l^−1^ calcium nitrate solution collection. The fibers of ∼2.2 mm in diameter after immersing for 30 min were cut and spilt into cylindrical granules whose lengths were approximately 1.5 mm. Then, the granules were dried and finally sintered at 1150 °C for 3 hours. The dual-core-type bioceramic granules with different shell components were denoted as P8-Sr4@Zn3, P8-Sr4@TCP, and P8-Sr4@HAR.

### 2.4 Basic characterizations of the dual-core-component bioceramic granules

Using an X'Pert PRO X-ray diffractometer (Holland) with a CuKα anode, the phases of the bioceramic powders were characterized from 10° to 60°/2θ in steps of 0.026°/2θ. The fracture surfaces of the dual-core-component granules were characterized by scanning electron microscopy (Gemini Sigma 300, Zeiss, Germany) with an accelerating voltage of 3 kV. Face-scanning energy dispersive spectroscopy (EDS) was used to evaluate the elemental composition of different components in the core and shell layers. The fracture-surface diameters of the granules were measured. Using a universal testing machine (Model 4502; Instron), the data of compressive tests for the sintered granules were measured at a strain rate of 20 N min^−1^ at ordinary temperature (*n* = 3). Then, the compressive stress–strain curves were collected using a software called Bluehill LE.

### 2.5 *In vitro* bio-dissolution analysis of the dual-core granules

To evaluate the bioactive inorganic ion release behavior of Mg^2+^, Sr^2+^, Ca^2+^, PO_4_^3−^ and SiO_3_^2−^ from the dual-core bioceramic granules (*W*_0_), the granules (1.0 g; *n* = 3) were submerged by 20 mL Tris buffer with an initial pH ∼7.40 at 37 °C. At 1, 3, 5, 7, 14, and 28 days, the Tris supernatant (1.0 mL) was centrifuged, followed by diluting with 5% HCl solution for ion determination using inductive coupled plasma atomic emission spectroscopy (ICP-AES; Thermo), and the Tris buffer was replenished to the original amount. These dual-core granules were dried up and weighed (*W*) once a week to evaluate the level of biodegradation *in vitro*. The mass loss was derived using the following formula: mass decrease = *W*/*W*_0_ × 100%.

### 2.6 Biomimetic re-mineralization evaluation *in vitro*

The biomimetic re-mineralization potential of the dual-core bioceramic granules was immersed in a famous simulated body fluid (SBF) medium *in vitro*. The granules (1.0 g; *n* = 3) were fully immersed in 10 mL SBF in a 37 °C incubator. The SBF was renewed every 2 days throughout the immersion period. After one and two weeks, the granules were rinsed three times with deionized water and absolute ethanol. The granule surface was observed using SEM, and the Ca/P ratio was determined by quantitative EDS measurement.

### 2.7 Critical size femoral bone defect model evaluation *in vivo*

Twenty-four male New Zealand rabbits (3.0–3.2 kg, 4 months old) were purchased for femoral condyle implantation experiments, which were randomly divided into three groups of 8 animals each for 6 and 18 week time points. The experiment was approved by the Ethics Committee of Zhejiang University (ZJU20220475).^20^ All the rabbits were generally anesthetized by auricular vein injection of sodium pentobarbital (1 mg kg^−1^, Sigma) in advance. Then, a critical-sized cylindrical defect (Ø ∼7.0 × 6.2 mm) was caused by dental trephine (Ø 7 mm) in the condylus lateralis femoris on each rabbit and filled some (23–25 in amount) dual-core granules (diameter: ∼1.7 mm; height: ∼1.6 mm) into the bone defects. The incision was treated for anti-infection by applying penicillin powder on the wound after implantation. Over the next 3 days, these animals received an intramuscular injection of penicillin daily to prevent them from infection. After that, rabbits were sacrificed by pentobarbital sodium(iv) at 6 and 18 weeks, and femoral bone specimens were collected and fixed in the paraformaldehyde fixation solution.

### 2.8 Micro-CT analysis and X-ray radiological examination

New bone tissue ingrowth into the defects was observed by X-rays (XPERT; KUBTEC Co., USA). The X-ray images of femoral bone specimens were taken at 200 μA and 45 kV to evaluate the mineral deposition qualitatively. Subsequently, with the Micro-CT imaging system (AX2000 CT scanner, Always Imaging, Shanghai), the distribution and growth of new bone tissue were assessed using Micro-CT. These specimens were scanned along the anatomical axis of the femur at ambient temperature. The X-rays originate from Microfocus X-ray Source (FineTec 160 kV, Germany) along with an X-ray detector (NDT 1717M; voxel size: 14 μm; pixel size: 139 μm), which help to generate continuous cross-sectional images (image resolution: 15.6 μm; time of exposure: 500 ms). The cylindrical region of interest (ROI; Ø 7.0 × 6.0 mm) was set surrounding the fracture zone, and the new bone tissue in the ROI was identified and distinguished from the biomaterials by analyzing quantitative images based on the density difference. The trabecular morphological parameters including bone volume fraction (BV/TV%), trabecular number (Tb.N; 1 mm^−1^) and residual volume/total volume (RV/TV%) were calculated using VG Studio MAX.

### 2.9 Histological progress

The granule-filled femoral bone specimens were incubated in 10% buffered formaldehyde for one week for fixation, followed by rinsing with pure water overnight. After dehydration in a graded series of ethanol (70–100%), the specimens were rinsed with Xylene. Then, they were embedded in the mixtures of polymethyl methacrylate (PMMA), bis(*tert*-butyldioxyisopropyl) benzene and dibutyl phthalate. Along the long axis of the femur using a saw microtome (Leica, SP1600, Nussloch, Germany), several rabbit specimens containing the bone tissue and implants were cut into 6–8 thin sections after polymerization. To prevent them from impurities and scratches, these thin sections were finely polished by applying the machine (MP-2B). After that, sections were divided into two parts and distributed for H&E and Masson trichrome staining.

### 2.10 Measurement data analysis

The measurement data were shown in the form of means ± SD (standard deviation); besides, some quantitative data were compared and analyzed using one-way analysis of variance (ANOVA), and a value of *p* < 0.05 was considered statistical significance.

## 3. Results and discussion

### 3.1 Primary characterization of the porous dual-core layer granules

According to the measurement data and ICP analysis results, the as-calcined bioceramic granules showed that the Zn/Ca, P/Si and Sr/Ca molar ratios were 3.50%, 3.87% and 7.85% for the CSi-Zn3, CSi-Sr4 and CSi-P8 respectively. Besides, the Zn/Ca ratio (3.50%) was slightly higher than the expected results. As expected, the dual-core-shell-typed bioceramic fibers could be made easily through 18G-18G@10G nozzles (Ø 0.8 mm, Ø 0.8 mm, Ø 2.50 mm in inner diameter) following the sintering treatment (Fig. 1A). In addition, it was obvious that bioceramic fibers with similar chemical component (*i.e.*, CSi) had a reduced diameter during the sintering period (Fig. 1B). The compressive tests are shown in Fig. 1C, and the stress–strain curves showed that the P8-Sr4@HAR granules demonstrated better compressive resistance. In addition, the β-TCP considerably weakened the compressive mechanical properties of the core–shell granules. The XRD patterns of the five parts of the dual-core-shell granules are illustrated in Fig. 1D–E. The β-TCP and HAR highly agreed with standard values (PDF#09-0169; PDF#35-0745), in which none of the discernable peaks was attributed to the other Ca-silicates. The main characteristic peaks at 25.83°, 27.87°, 31.05° and 34.27°/2*θ* were assigned to the β-TCP structure. Additionally, the results indicated that the main characteristic peaks of the HAR structure were at 23.89°, 28.93°, 31.11° and 36.23°/2*θ*. For the XRD results for CSi core components (CSi-Zn4, CSi-P8, CSi-Sr4), which were foreign ion-doped, the peaks at 27.86° and 29.88°/2*θ* could be attributed to the phase of CSi, which confirmed that the original crystalline phase was preserved in the core-layer components.

An ideal bio-material should provide sufficient strength support for the defect site and new bone tissue in the process of bone repair, which requires the material to have mechanical properties that match the bone tissue. Among the three materials, P8-Sr4@HAR showed better mechanical properties. Some studies also pointed out that HAR has good biocompatibility, compressive strength and fracture toughness.^32^ However, the poor mechanical properties of β-TCP may be due to the β-α phase conversion of β-TCP powder at 1115–1150 °C,^33,34^ which can lead to the generation of cracks.^35–37^ Because the phase transition temperature was carried out at a lower temperature, this was also not conducive to the generation of high-density materials. Therefore, it is difficult to improve the overall strength and mechanical properties of β-TCP through the sintering process.

### 3.2 FE-SEM/EDS analysis of the porous dual-core layer granules

**Fig. 2:**
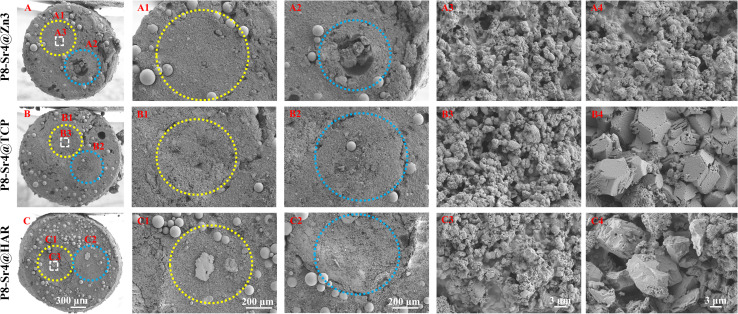
Sem images of the dual-core-type granules before sintering. (A–A4) P8-Sr4@Zn3; (B–B4) P8-Sr4@TCP; (C–C4) P8-Sr4@HAR. The yellow and blue circles are the dual-core component (P8-Sr4) before sintering.

**Fig. 3:**
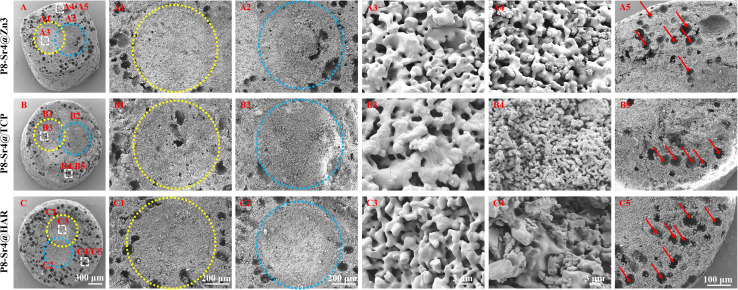
Sem images of the porous dual-core-type granules after sintering. (A–A5) P8-Sr4@Zn3; (B–B5) P8-Sr4@Tcp; (C–C5) P8-Sr4@Har. The yellow and blue circles are the dual-core component (P8-Sr4) after sintering. The red arrows indicate the pore structures left by PMMA.

**Fig. 4:**
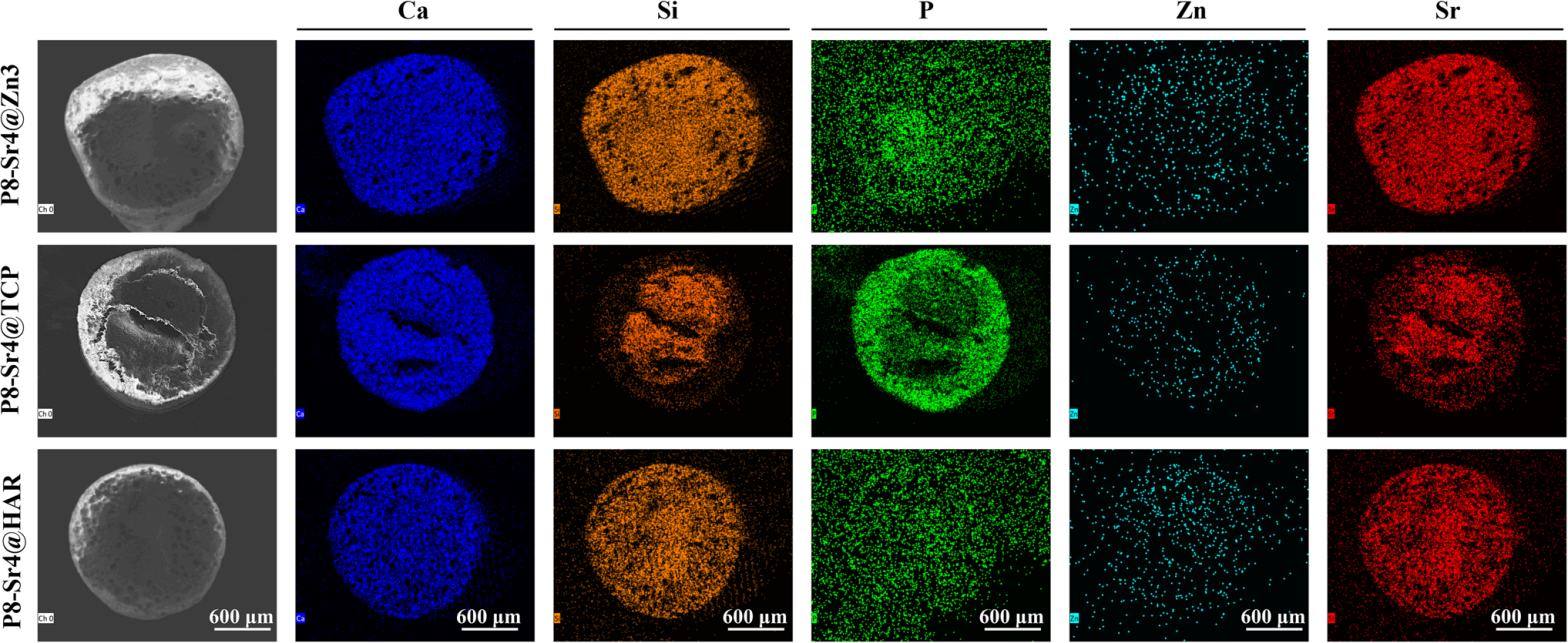
Composition analysis *via* EDS mapping for the porous dual-core-type bioceramic granules.

SEM observation was used to confirm the component distribution of the porous dual-core layer granules (before sintering). As shown in Fig. 2, the SEM images (×50 magnification) indicated the distinct porous dual-core structures in the fracture surface of the samples. In addition, the stray distribution of the PMMA microspheres was easily observed, and the microspheres were found only in the shell regions of the samples. Moreover, it is easy to identify the circular dividing line between the two core layers and the shell region, and there is no contact between the core layer regions before sintering. As shown in Fig. 3, the dual-core layer region overlap was easily observed, indicating that the diffusion of the phase components occurred between the dual-core layer regions during sintering. The high-magnification diagram (×3000) shows the morphological differences between the three shell phase components before and after sintering. The particle size of CSi-Zn3 before sintering was the smallest, and the particles were similar to short rods. The β-TCP particles had a regular pentagonal shape, whose surface appeared flatter. The shape of the HAR particles was lumpy, the particle size was relatively coarse, and the arrangement was relatively messy. After sintering, the CSi component in these particles showed a mesh arrangement, and its structure was looser than that of the other two components. The β-TCP original regular polyhedral structure was transformed into granular and short rod-like accumulation after sintering. The originally dispersed particles of HAR were connected, whose degree of binding changed most obviously. In addition, the original PMMA disappears and leaves pores *in situ*. Face-scanning EDS analysis was employed to determine the elemental distribution on the surface of the porous dual-core layer granules to distinguish the different chemical compositions (Fig. 4). In P8-Sr4@TCP particles, phosphorus (P) was mainly distributed in the periphery of the particles, while silicon (Si) was mainly accumulated in the dual-core layer, which was consistent with the SEM results, and strontium (Sr) could be detected in both core regions owing to the diffusion of both core phases. For the P8-Sr4@Zn3 and P8-Sr4@HAR particles, the overall element distribution is also consistent with the SEM structure. There was phase diffusion in the sintering process of the two core layer structures, and the three shell components all underwent large structural changes, among which the HAR structure appeared to be more compact.

### 3.3 Bio-dissolution of the porous dual-core layer granules *in vitro*

**Fig. 5:**
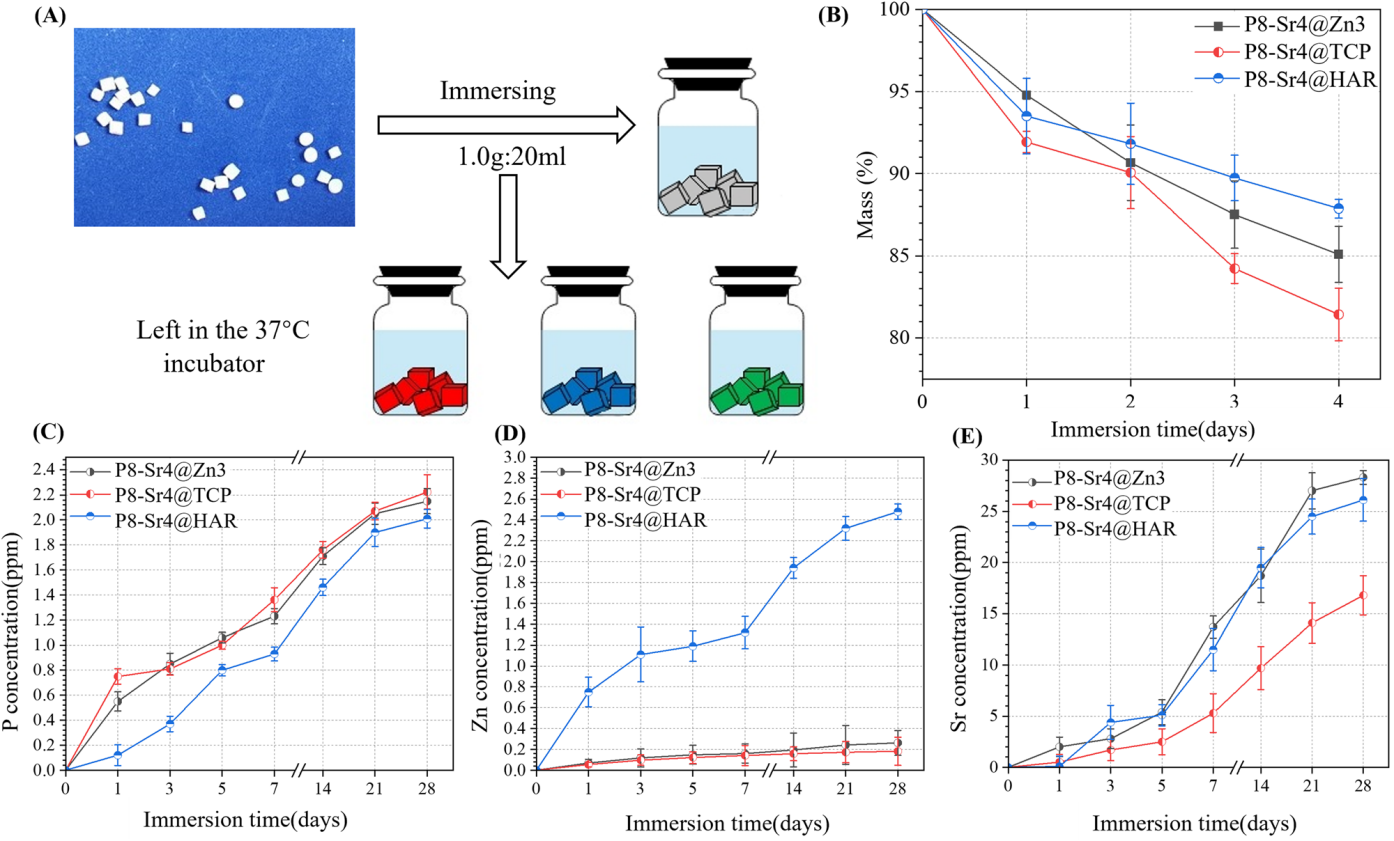
Bio-dissolution evaluation in Tris buffer *in vitro*. (A) Schematic diagram of the ion release test; (B) mass decay of the bioceramic granules with time; (C–E) changes in P, Zn, and Sr concentrations in the Tris buffer with time.

To stimulate the biodegradation process *in vivo*, the porous dual-core layer granules were submerged in Tris buffer (Fig. 5A). To measure the bio-dissolution ability of porous dual-core layer granules, mass loss curves were established, which showed that the minimum slope contributed to the P8-Sr4@HAR. The result reflects that HAR is the least prone among the three granules (Fig. 5B). The mass of P8-Sr4@HAR lost 12.13% after 28 days, while the corresponding mass loss of P8-Sr4@Zn3 (14.91%) and P8-Sr4@TCP (18.57%) granules increased sequentially. Besides, β-TCP showed good biological solubility in the result, which was similar to the results of some studies,^38,39^ but others reported the slow dissolution of β-TCP,^40,41^ which may be due to the volume/kinetic effect^42,43^ and the implantation site *in vivo*. In addition, the biological solubility of HAR was poor, as expected by the assay.

The concentration curves of phosphorus (P), zinc (Zn) and strontium (Sr) ions are shown in Fig. 5C–E. The release rates of the P8-Sr4@TCP groups were the fastest because both shell and core can release phosphorus ions, and the remaining two groups also had good phosphorus release ability, indicating that the phosphorus released during the immersion period was mainly from the CSi-P8 component of the nuclear layer. Phosphorus played an important role in physiological processes, such as cell signal transduction, energy metabolism and acid-base balance. It may actively regulate the proliferation and differentiation of osteoblasts and pre-osteoblasts through the ERK1/2 signaling pathway and positively regulate cell apoptosis by reducing mitochondrial transmembrane potential.^44^ Additionally, among the three groups, the P8-Sr4@HAR particles had the highest zinc ion dissolution rate because the composition and porous structure of CSi in the nuclear layer improved the biological solubility of HAR. As an important component of the enzyme system that affects cell division and proliferation, zinc is involved in the regulation of cell proliferation in various ways.^45^ Therefore, compared with wollastonite, HAR showed a more significant promotion effect on cell proliferation and differentiation.^46^ In addition, zinc feldspar has excellent anti-bacterial properties. Li's studies introduced a zinc-jaundrite coating on the Ti-6Al-4V matrix by plasma spraying, which showed a 93% antibacterial rate against *Staphylococcus aureus*.^47^ The results suggest that CSi improves the release rate of zinc in HAR material and enhances the biological properties. In addition, although the P8-Sr4@TCP particles had the fastest rate of degradation, they had the weakest ability to release strontium ions outward, which implies that the shell layer greatly affects the ion release behavior of the core layer. Strontium plays a dual role in bone metabolism by stimulating bone formation and inhibiting bone resorption mainly by regulating alkaline phosphatase (ALP) activity in osteoblasts.^48–50^

Therefore, phosphorus, zinc and strontium ions are beneficial to stimulate bone formation in biomaterial preparation and can be selectively incorporated into some bioceramic implants.

### 3.4 *In vitro* bioactivity (apatite mineralization) evaluation

**Fig. 6:**
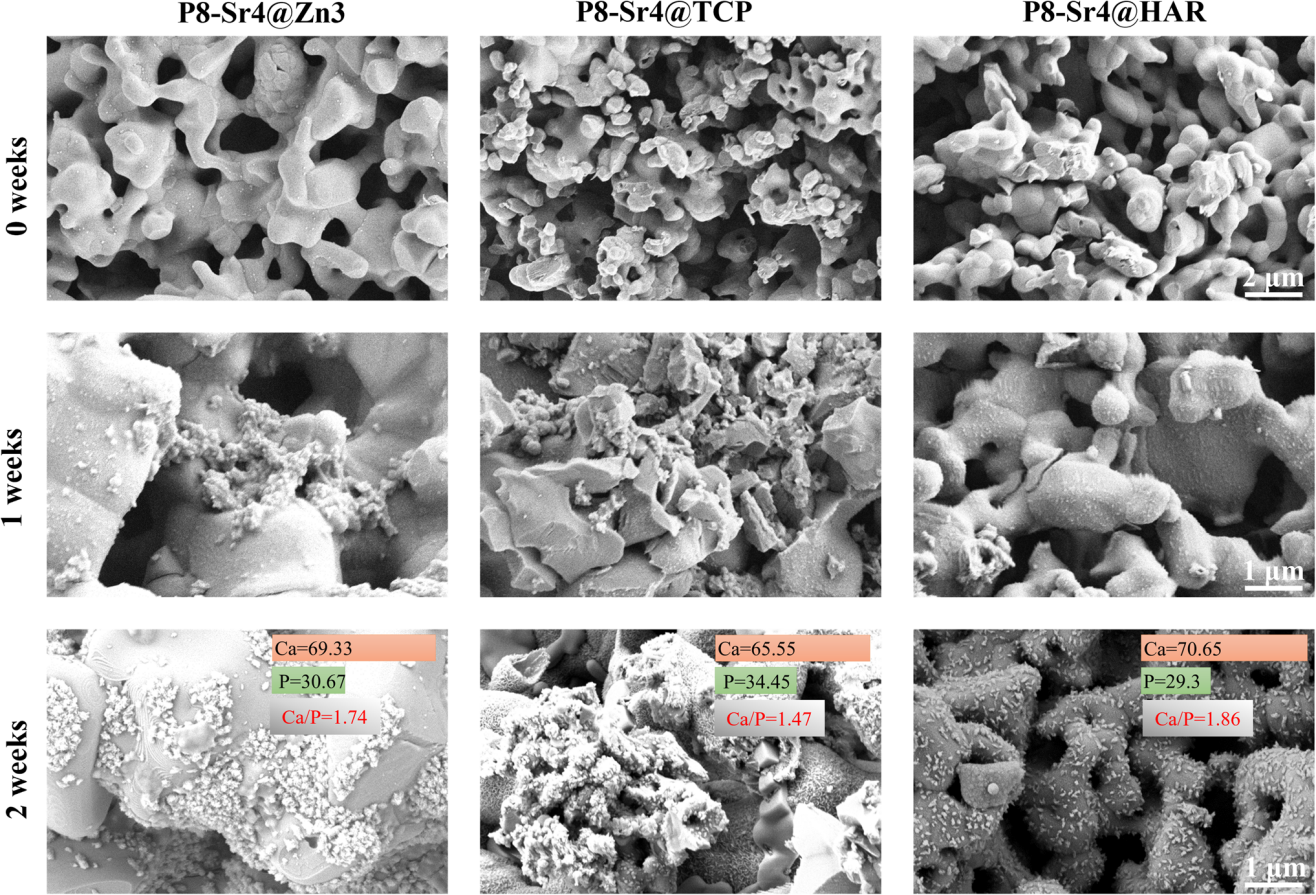
Sem images of dual-core-type bioceramic granules after immersion in sbf (1.0 g/10 ml) for 0, 1 and 2 weeks.

The three groups of granule samples were submerged in the SBF for one and two weeks; then, the surface images were taken by SEM to observe and analyse the newly formed apatite. Some apatite-like substances were accumulating on the surface of the immersed granules in the form of microspheres in high magnification (×10 000) SEM images (Fig. 6). One week after the immersion, the apatite-like grains were found in both P8-Sr4@Zn3 and P8-Sr4@TCP groups clearly, but not in P8-Sr4@HAR groups. At two weeks, on the surface of the three immersed granules, a large amount of apatite-like microsphere substances was observed. Besides, the P8-Sr4@Zn3 group had the widest coverage, while the P8-Sr4@TCP had the densest accumulation degree but was not as wide. Additionally, the number of apatite-like substances on the surface of P8-Sr4@HAR was relatively the least. Through the quantitative EDS analysis, the Ca/P ratios in the surface layer of the granules were P8-Sr4@Zn3 (1.74), P8-Sr4@TCP (1.45) and P8-Sr4@HAR (1.86), which were close to hydroxyapatite (1.67).

Bioactivity has traditionally been considered the decisive factor in promoting the fixation of implants with bone tissue and is ultimately the key to the success of bone grafting materials. It is generally accepted that the biological activity of artificial materials *in vivo* can be largely predicted by the formation of a bionic appetite after immersion in SBF.^51^ The results of SEM images and EDS analysis confirmed that all three groups of the porous dual-core layer granules can significantly induce the formation of apatite-like layers, which would benefit the following integration between the material and bone. The P8-Sr4@TCP granules had better bioactivity capacity than the others.

### 3.5 Systematic characterization of the porous dual-core layer granules in bone defects

#### 3.5.1 Radiological examination by X-ray scanning

**Fig. 7:**
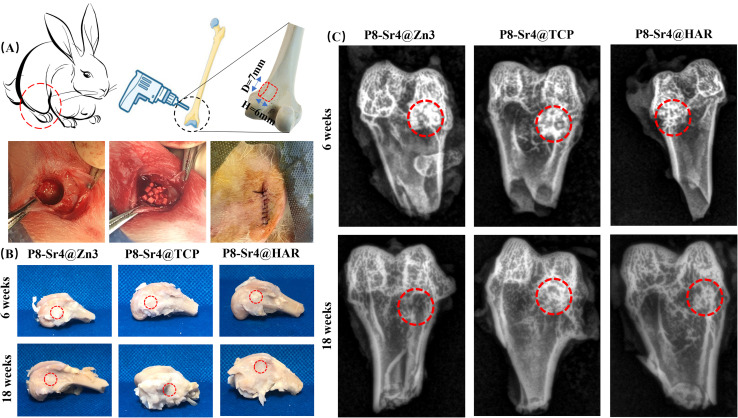
Animal model and primary analyses of the bone repair with the dual-core-type bioceramic granules. (A) Schematic illustration of the bone defect model and bioceramic granules implantation in femoral bone defects; (B) the harvested femoral bone specimens at 6 and 18 weeks. (C) X-ray images of femoral bone defects filled with the bioceramic granules after implantation for 6 and 18 weeks.

The animal model pattern of the implantation of the porous dual-core layer granules is demonstrated in Fig. 7A. The roentgenographic analysis indicated that the granules were implanted in the expected position, and the lower X radiodensity revealed the formation of new bone (Fig. 7C). At 6 weeks, the difference in X radiodensity between the three groups was not clear, which revealed that the rate of biodegradation was similar in the three granules. At 18 weeks, the P8-Sr4@Zn3 granules were difficult to observe in the expected position, indicating the high level of granule degradation in bone defects. Besides, the X radiodensity of the other groups became lower compared to the results of 6 weeks, but the remnant granules could still be found.

#### 3.5.2 μCT analysis

**Fig. 8:**
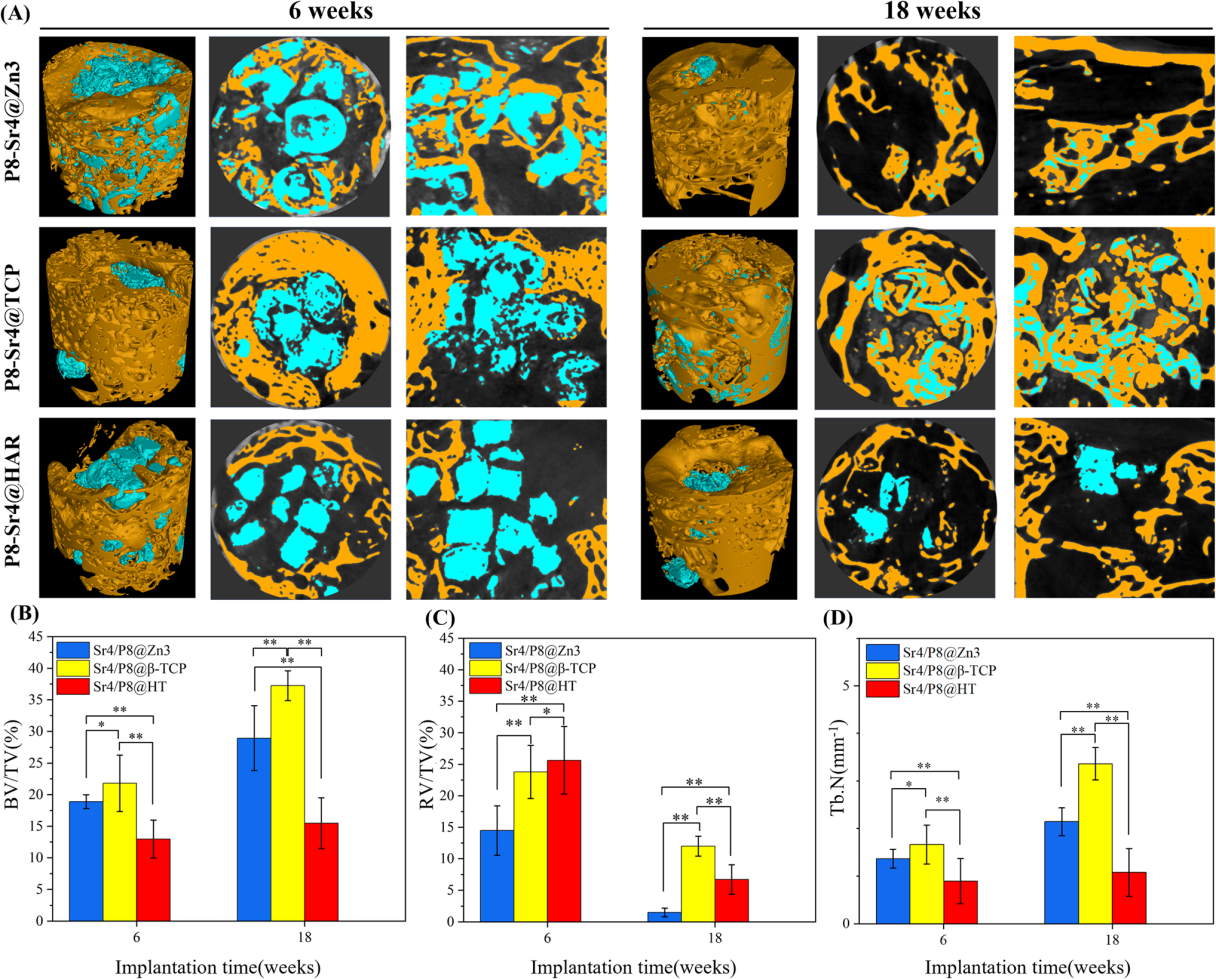
2D/3D μCT reconstruction and quantitative analyses of femoral bone defects filled with the dual-core-type bioceramic granules after implantation for 6 and 18 weeks. (A) μCT images; (B) BV/TV (bone volume/total volume); (C) RV/TV (residual volume/total volume); (D) Tb.N (trabecular number). *p < 0.05; **p < 0.01.

The 2D/3D μCT analysis was performed at 6 and 18 weeks post-implantation to evaluate and observe the repair process of the femoral bone (Fig. 8A). At 6 weeks, it was easy to observe that the P8-Sr4@Zn3 granules were significantly degraded and new bone actively penetrated the material space, which means that the connection of new bone contact was achieved through binding to the material throughout the defect space. Additionally, the P8-Sr4@TCP granules lost their original cylindrical core–shell structure within a short time after implantation. A large number of new bone tissue appeared surrounding the broken granules, which showed an ingrown trend. In addition, the P8-Sr4@HAR granules showed the lowest degree of degradation, while some new bone formations occurred surrounding the granules. At 18 weeks, the result showed that most P8-Sr4@Zn3 granules were degraded completely, and the few remaining parts were tightly integrated with new bone tissue; the P8-Sr4@TCP granules still had a considerable amount of residual that was mainly the shell part. The remaining parts were tightly close to the new bone, and the bone growth into the material could also be observed. The P8-Sr4@HAR granules also had a very obvious degradation; the new bone in the center of the defect area was less than that of the other two groups of materials, and there was no sign of bone ingression.

The trabecular morphological parameters are shown in Fig. 8B–D. These quantitative data were consistent with the μCT images, in which the trabecular number and bone volume fraction of the P8-Sr4@TCP group showed a significant difference from the other groups. The P8-Sr4@TCP granules had the highest bone volume fraction at 6 and 18 weeks, which were 21.81% and 37.23%, respectively. The excellent ability of osteogenesis was also reflected by the highest trabecular numbers of 1.66 mm^−1^ and 3.36 mm^−1^ at 6 and 18 weeks, respectively, indicating the best bonding between the material and bone. The remaining material volume fraction of P8-Sr4@TCP granules was the highest among the three groups at 18 weeks (12.00%); the P8-Sr4@HAR granules ranked second (6.71%), and the P8-Sr4@Zn3 granules were the smallest (1.51%), which implied that most of the material parts were completely degraded.

#### 3.5.3 Histological progress

**Fig. 9:**
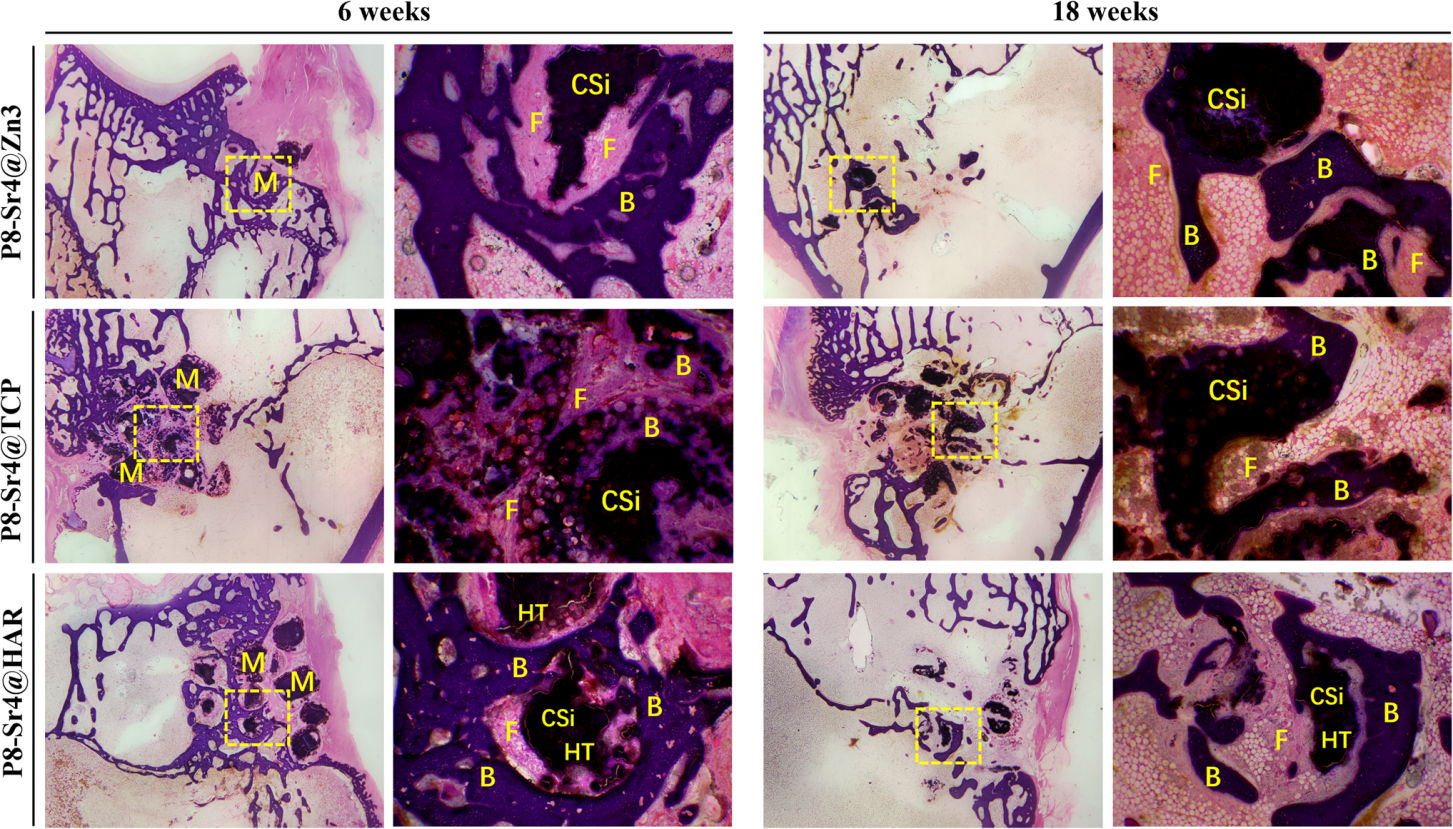
H&E staining histological evaluation (10×, 40×) of bone regeneration for three groups of dual-core-type bioceramic granules at 6 and 18 weeks. B: bone; F: fibrous tissue; CSi: wollastonite core; HAR: hardystonite (HT); M: materials (granules).

**Fig. 10:**
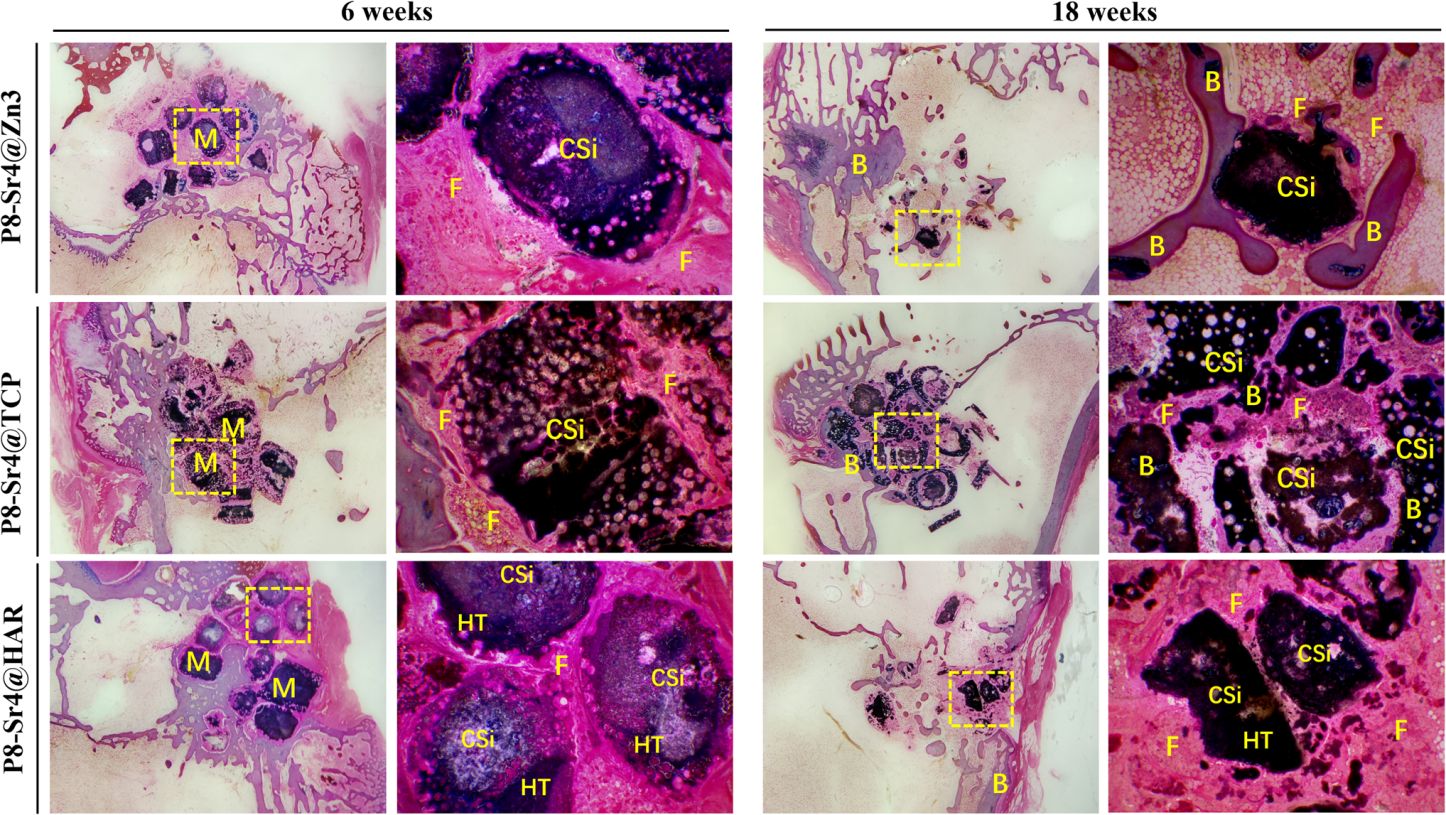
Masson's trichome staining histological evaluation (10×, 40×) of bone regeneration for three groups of dual-core-type bioceramic granules at 6 and 18 weeks. B: bone; F: fibrous tissue; CSi: wollastonite core; HAR: hardystonite (HT); M: materials (granules).

Fig. 9 and 10 show the results of the implantation of dual-core porous biological ceramic particles and the bone interface layer of HE and Masson staining histological appearance after 6 and 18 weeks. At 6 weeks, the outer shell structure of the P8-Sr4@Zn3 particles was degraded, and a large amount of fibrous tissue and a small amount of new bone tissue were distributed around it. The overall structure of P8-Sr4@TCP particles showed a large number of vacuolar destruction, and interstitial fibrous tissue and new bone tissue distribution were observed. The P8-Sr4@HAR core layer degraded faster than the shell HAR structure, and it was surrounded by a large amount of fibrous tissue, but no new bone tissue was found. After 18 weeks, the P8-Sr4@Zn3 particles were almost completely degraded due to their great degradation ability, and a large area of new bone was observed in the area where the original particles were located. Under high magnification (×40), the new bone tissue could be seen around the residual particles. The structure of P8-Sr4@TCP particles was seriously broken, and a large number of new bones penetrated through the intergranular space. The boundary was unclear, and it was difficult to distinguish the P8-Sr4@TCP particles and new bone tissue under high magnification (×40). There was little new bone tissue around the P8-Sr4@HAR granules; the boundary was clear, and there was a small amount of fibrous tissue in the void.

In general, the results of animal critical size femoral bone defect model experiments showed the differences between the three materials after implantation. Among them, P8-Sr4@Zn3 particles exhibited the fastest degradation rate *in vivo*, which showed the best bone regeneration effect among the three materials. Its bone volume fraction was 37.23% and its trabecular number was 3.36 mm^−1^ at 18 weeks, which were significantly higher than those of other materials, indicating its good osteoinduction, which was consistent with the high phosphorus release rate of β-TCP. However, the remaining material volume fraction of P8-Sr4@TCP particles was also the highest, which was different from its results in the Tris immersion solution, presumably due to the influence of cell activity, pH value and other factors in the microenvironment of bone injury, and also reflected the unpredictable biodegradability of β-TCP. Although the composite of CSi and HAR can improve the degradation rate of P8-Sr4@HAR and show certain biological activity, there is still a significant gap between its ability to induce bone formation and P8-Sr4@TCP.

Overall, P8-Sr4@TCP showed excellent bone repair ability despite the degradation rate and mechanical properties defects, indicating that Sr4/P8@β-TCP particles were suitable for bone regeneration and repair in sites with low load bearing and antibacterial requirements. Considering the slow degradation rate, it is necessary to optimize and regulate the degradation process to prevent an inflammatory response. Besides, P8-Sr4@Zn3 particles showed the prominent ability of degradation, which required coordination of degradation with the growth process of bone tissue. Additionally, P8-Sr4@HAR particles have poor osteoconductive ability, but the dual-core structure can significantly improve the degradation of the particles. Simultaneously, it has good zinc ion release ability and compressive strength, which can have more bearing and antibacterial potentials. Obviously, how to improve the biological activity of the HAR complex needs to be further explored.

## 4. Conclusion

In summary, multicomponent bioceramic fibrous particles doped with various bioactive ions were successfully fabricated in this study. Doping minor amounts of exogenous ions, such as phosphorus, zinc and strontium, can enhance the ability to release active ions and improve the resultant new bone formation. Among them, P8-Sr4@HAR particles significantly improved the biodegradation rate and osteogenic activity of pure HAR particles. Besides, despite deficient mechanical properties, P8-Sr4@TCP particles exhibited the most appreciable osteogenic capacity and expected biodegradation, which was mostly favorable for bone repair in critical bone defects. Therefore, the superior ability of osteo-stimulation combined with greatly enhanced biological activity demonstrates that P8-Sr4@TCP dual-core shell fibrous granules will play an important role and be promising implants for bone defect repair.

## Author contributions

Binji Cao, Yan Xu and Zhaonan Bao: materials preparation, sample testing, statistics collection and analysis, article writing; Yan Zhang, Yingjie Wang and Lijun Xie: *in vivo* trials and specimen collection; Yan Xu, Jian Shen and Yingjie Wang: proofread the paper; Xianyan Yang and Xisheng Weng: technical guidance; Cong Wang and Zhongru Gou: financial support, experimental proposal and writing guidance.

## Conflicts of interest

The authors declare that there is no conflict of interest.

## Supplementary Material
